# Case of a Dropsical Enlargement of the Abdomen

**Published:** 1804-09-01

**Authors:** John Pulley

**Affiliations:** Bedford


### Case of a Dropsical Enlargement of the Abdomen; *communicated by* Mr. John Pulley, *of Bedford.*

GEORGE BERRIDGE, a healthy lad, eighteen years of age, was lately admitted into the Bedford Infirmary with a dropsical enlargement of the abdomen. Two years and upward upward since, he received a kick from a horse on the left hypochondriac region, which was attended for some time with pain, and very speedily succeeded by a sensible enlargement of the body. At the time of the accident he had no surgical assistance, and suffered but little injury in his health. The swelling of the abdomen went on increasing to the period of his admission into the infirmary, unaccompanied by symptoms of irritation, or any apparent deviation (excepting the tumour, and a slight pain occasionally about the epigastric region) from a state of perfect health: his appetite was good; his chyle formed well in quantity and quality, as digestion went on without anxiety, and he lost no flesh; his pulse was regular, and respiration uninterrupted; and his excretions in every respect natural. During his residence in the hospital, he took such medicines, under the direction of his physician, as appeared likely to give a chance for the removal of the swelling, but without effect; therefore it was determined to have recourse to the trocar on the 24th of this month. It ought to be remarked here, that the swelling of the abdomen was uniform in appearance, and that the touch received an uniform sensation of fluctuation, not more or less evident in one part than another. On the day appointed, I divided the integuments with a lancet about an inch and a half below the umbilicus, in the direction of the linea alba, and then thrust the trocar into the abdomen, which penetrated with more than usual difficulty, as the integuments receded much inward. On withdrawing the trocar, the fluid issuing through the canula in a very interrupted stream, a probe was introduced, when several portions of ruptured hydatid vesicles were emitted with the aqueous matter. By this time about three quarters of a pint of yellowish fluid were discharged; and it being then very evident that the dropsical affection was encysted, the canula was withdrawn, and the lips of the wound brought together as speedily as possible. The lad was put to bed without suffering from pain or faintness; but in a few hours he was attacked by peritoneal inflammation, and died on the following day, about five in the afternoon. The next morning, in conjunction with the professional gentlemen of the infirmary, I opened his body, and the appearances on dissection, though not singular with regard to the disease, were peculiar in extent, and therefore seem well worthy of remark.

DISSECTION.

### Dissection.

On dividing the integuments downward from the ensiform cartilage, the peritoneum was incised, when a yellowish fluid burst out; and on prosecuting the incision, it was copiously evacuated. The necessary division of parts being made, a large cyst, capable of containing full two gallons of water, was discovered; it adhered firmly to the anterior part of the peritoneum, to the liver, spleen, and omentum; it was, in every respect, a perfect sac, without the *direct* aid of the peritonem, and occupied the greater part of the cavity of the abdomen. Within this cyst, hydatids were contained to the amount of several hundreds, floating, globular in form, and varying in size, from the bigness of a small pea to the magnitude of a hen's egg. Without this sac, but adhering to it, in the left hypochondriac region, and on the under side of the spleen, were situated two other hydatids of considerable size; they were not in contact with each other, but adhered firmly to the spleen, which viscus appeared diminished in its usual magnitude, as it was much expanded on the surface of these cysts, probably owing to compression. Another hydatid, containing not less than half a pint of fluid, but which, when removed from the body, was accidentally punctured, was situated above the spleen, and adhered firmly to the diaphragm. In the right hypochondriac region, between the larger lobe of the liver and the diaphragm, and firmly adhering to them, another hydatid was found, irregular in form, and about the size of a small orange. Under the liver, between the lobula Spigelii and the gall-bladder, was placed another hydatid about the size of a hen's egg, much indented about its centre, and assuming that appearance which we should suppose the uterus to obtain in its hour-glass contraction. Another hydatid, similar in form, but somewhat less in size, was attached to the peritoneal covering of the colon near to its sigmoid flexure. The last hydatid to be mentioned was situated chiefly within the pelvis, between the urinary bladder and the rectum; it contained not less than half a pint of fluid, and took much the form of a child's head on its egress from the pelvis. The circumference of this hydatid measured ten inches and a quarter, and from point to point, eleven inches and a half. It is singular, that this tumour occasioned no interruption to the evacuation of urine or fæces. The larger hydatid adhering to the spleen, measured, in its body, ten inches and three quarters, quarters, and from point to point, twelve inches and a quarter; the other was not much inferior in size. Throughout the dissection, there appeared no visceral disease. In the thorax no hydatid was contained, and the heart and lungs maintained a healthy aspect. In the abdomen, those hydatids connected with the liver, were attached to its peritoneal covering, without arising from or interfering with its substance. Perhaps it may be conjectured, that those hydatids attached to the spleen, arose within its substance. Most of the smaller hydatids were very transparent, containing a fluid " limpid as the crystal stream,' and not coagulable.

I am not aware that any case of hydatid-formation has been offered to the public so extensive as the present; but a case, very nearly allied, is related by Cheselden, in his Anatomy of the Human Body, published in the year 1713.

Whatever may be the prevailing opinion concerning the formation of hydatids, it would appear, in this instance, that they took their origin from the accident. Inflammation of the peritoneum, doubtless, was the consequence of the kick, and probably an effusion of coagulable lymph, the birth of cystic dropsy. The largest sac was connected with the part that received the inflicted blow; most probably, it was the first formed, and from its rapid increase, possibly it assumed an inflammatory state, and by an effusion of coagulable lymph from its inner surface, might give rise to the existence of those minor hydatids, which were found floating within its cavity. This sac possessed much firmness and density, and certainly was highly vascular.

